# Newly emerged ROS1 rearrangement in a patient with lung adenocarcinoma following resistance to immune checkpoint inhibitors: a case report

**DOI:** 10.3389/fonc.2024.1507658

**Published:** 2024-12-11

**Authors:** Jian Wang, Bingyue Liu, Qinhong Zheng, Ruoshui Xiao, Jianxin Chen

**Affiliations:** ^1^ Department of Medical Oncology, International Ward, The Quzhou Affiliated Hospital of Wenzhou Medical University, Quzhou People’s Hospital, Quzhou, China; ^2^ Jinhua Joint Training Base, The Third Clinical Medical College of Zhejiang Chinese Medical University, Hangzhou, China; ^3^ Department of Orthopaedics, Hangzhou Zhanshi Traditional Chinese Hospital of Orthopaedics, Hangzhou, China

**Keywords:** ROS-1 rearrangement, lung adenocarcinoma, resistance, immune checkpoint inhibitors, pembrolizumab, case report

## Abstract

**Background:**

ROS1, a member of the sevenless subfamily of tyrosine kinase insulin receptors, promotes tumor cell survival, proliferation, and metastasis by activating the JAK/STAT, PI3K/AKT, and MAPK/ERK pathways. It only accounts for about 2% of total NSCLC cases. No cases of acquired ROS-1 rearrangement have been reported worldwide.

**Case presentation:**

We reported a case of lung adenocarcinoma without driver alteration that developed resistance to pembrolizumab and newly emerged CD74-ROS1 fusion, and achieved a partial response after entrectinib treatment.

**Conclusions:**

We hypothesize that the newly emerged ROS1 rearrangement occurs as the subset of cells harboring ROS1 gradually becomes the predominant pathological type of adenocarcinoma following pembrolizumab treatment. We propose that new therapeutic targets may emerge for this patient population following long-term immunotherapy. Thus, we advocate for regular monitoring of tumor genetic status, which could yield unexpected benefits.

## Highlights

- It is the first report of newly emerged ROS1 rearrangement after resistance to immune checkpoint inhibitors in clinical practice of NSCLC.
- This study suggests that there is a possibility that new therapeutic targets may emerge in patients during long-term immunotherapy.
- We advocate for regular monitoring and retesting of driver genes in lung adenocarcinoma cases that lack driver alterations and exhibit resistance to immune checkpoint inhibitors, as it could yield unexpected benefits.

## 1 Introduction

Lung cancer, a significant global public health burden, is currently the leading cause of cancer-related deaths (1, 2). In 2023, the American Cancer Society estimates that there will be 238,340 new cases and 127,070 lung cancer-related deaths in the United States (2). Non-small cell lung cancer (NSCLC) is the most prevalent type, representing approximately 85% of all lung cancer, with more than half of NSCLC patients diagnosed at an advanced stage (IIIB/C or IV) (3, 4). Furthermore, two-thirds of Asian patients with advanced NSCLC present with driver genes, including KRAS, BRAF, EGFR, MET, and ERBB2 mutations, as well as genomic rearrangements involving ALK, ROS1, RET, and NTRK (5, 6). Currently, most patients harboring these mutations can achieve varying degrees of response through targeted therapies. Consequently, the National Comprehensive Cancer Network (NCCN) guidelines recommend molecular testing for established cancer driver genes in patients with advanced or metastatic NSCLC (7). For advanced NSCLC patients without targetable oncogenes, the ASCO guidelines recommend immune checkpoint inhibitors (ICIs) monotherapy or combined with chemotherapy as first-line treatment, achieving a five-year survival rate of 20%-30% (8).

ROS proto-oncogene 1, receptor tyrosine kinase (ROS1), located on chromosome 6q22.1, is a member of the sevenless subfamily of tyrosine kinase insulin receptors. The activation of ROS1 kinase leads to the upregulation of the JAK/STAT, PI3K/AKT, and MAPK/ERK signaling pathways, which play crucial roles in tumor cell survival, proliferation, and metastasis (9). There are approximately 1 to 2% NSCLC patients harboring ROS1 rearrangement (3). To date, there have been no reported cases of NSCLCL with secondary ROS1 rearrangement following immunotherapy worldwide. In this report, we present a case of lung adenocarcinoma lacking known cancer driver genes, which developed a ROS1 rearrangement after multiple cycles of pembrolizumab treatment and subsequently responded partially to entrectinib. We hope this case presentation could offer new insights for clinicians managing NSCLC patients who experience progression after immunotherapy.

## 2 Case presentation

**Figure 1:**
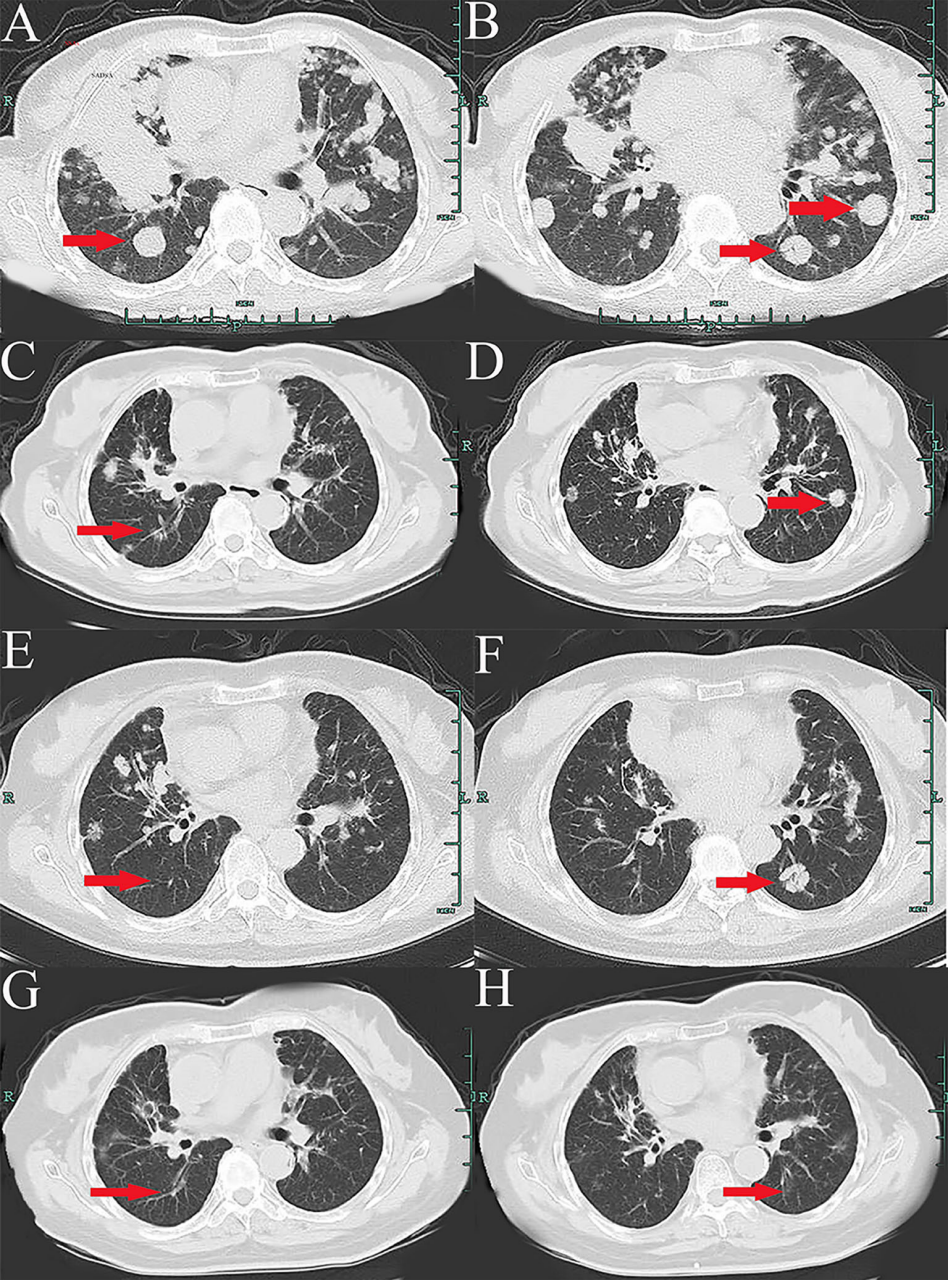
Variations of occupation on mediastinum and both hilar areas by CT images during the treatment (red arrowheads). **(A, B)**, February, 2022. **(C, D)**, March, 2023. **(E, F)**, April, 2024. **(G, H)**, July, 2024.

**Figure 2:**
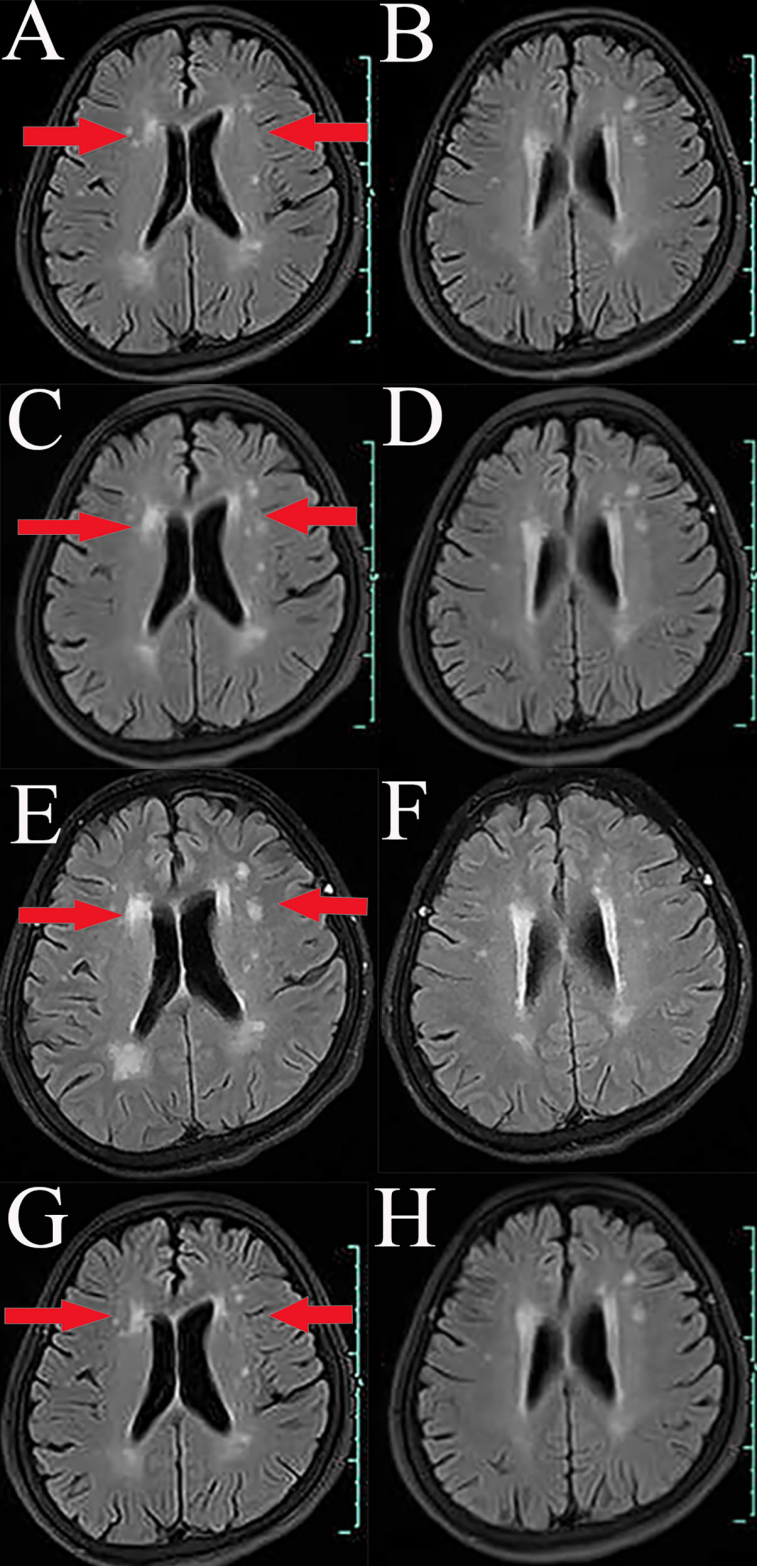
Variations of occupation on brain areas by MRI images during the treatment (red arrowheads). **(A, B)**, February, 2022. **(C, D)**, February, 2023. **(E, F)**, April, 2024. **(G, H)**, July, 2024.

**Figure 3:**
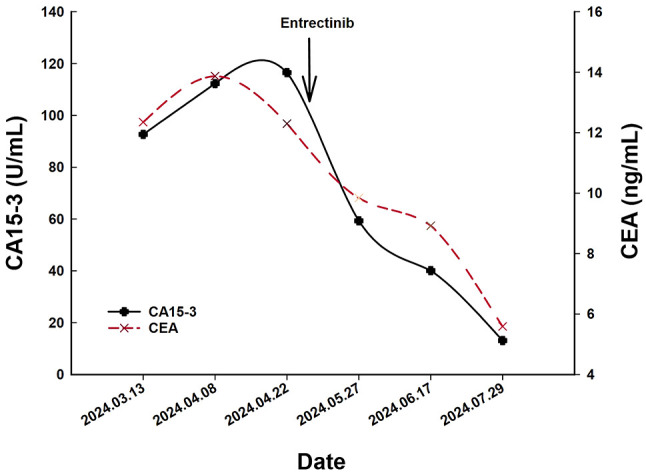
The variations of tumor marker CEA (normal range 0 to 5 ng/mL) and CA15-3 (normal range 0 to 25 U/mL) from March 13th to July 29th, 2024.

**Figure 4:**
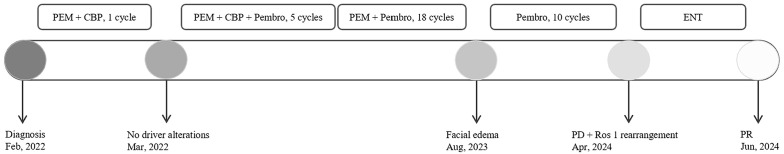
The overview of the approaches to diagnosis, and treatment regimens for each regimen. CBP, carboplatin; PEM, pemetrexed; Pembro, pembrolizumab; ENT, Entrectinib; PD, Progressive Disease; PR, partial response.

A 66-year-old Chinese woman was admitted to Quzhou People′s Hospital on February 18th, 2022 with a mild cough and sputum that had persisted for half a year. The patient denied any history of smoking, alcohol consumption, or any related medical or family history. Subsequent chest-enhanced computed tomography (CT) results revealed diffuse nodular shadows in both lungs, accompanied by multiple lymphadenopathies in the mediastinum (**Figures 1A, B**). A percutaneous lung biopsy was performed, and tissue samples obtained were subjected to immunohistochemical analysis, confirming a diagnosis of lung adenocarcinoma. An enhanced magnetic resonance imaging (MRI) of the head conducted on February 20th, 2022, demonstrated multiple spotty enhancement shadows in the brain (**Figures 2A, B**). Immunohistochemistry findings were as follows: CK7 (positive), CK20 (negative), CDX-2 (negative), TTF-1 (positive), Napsin A (positive); Ki-67 (positive, 5%), P40 (negative), SP-A (positive), S-100 (negative), and PD-L1 expression of <1%. Based on these results, a diagnosis of lung adenocarcinoma was made and staged as T4N2M1 according to the American Joint Committee on Cancer (AJCC) version 8th. The results of the Next-Generation Sequencing (NGS) test were not yet available when treatment decisions needed to be made. Considering the advanced stage of the disease, the patient was administered a regimen of pemetrexed 700mg and carboplatin 300 mg on February 25th, 2022. Subsequently, NGS did not identify any therapeutic targets, leading to the administration of five cycles of combined treatment with pembrolizumab (IV 240 mg on day 1, every 21 days), carboplatin (IV 400 mg on day 1, every 21 days), and pemetrexed (IV 700 mg on day 1, every 21 days), starting on March 18th, 2022. The patient then continued with 18 cycles of pembrolizumab (IV 240 mg on day 1, every 21 days) and pemetrexed (IV 700 mg on day 1, every 21 days) beginning July 1st, 2022. The brain MRI on February 21st, 2023 showed no progression (**Figure 2C, D**), and the chest CT on March 15th, 2023 showed that the mass was significantly smaller (**Figure 1C, D**). Due to the development of moderate facial edema on August 5th, 2023, we discontinued pemetrexed while continuing pembrolizumab for ten cycles. A follow-up chest CT (**Figures 1E, F**) and brain MRI (**Figures 2E, F**) on April 22nd, 2024, indicated that the tumor was slowly progressing. Subsequently, we performed NGS for the patient again, and the results showed the presence of a CD74-ROS1 fusion, with no variant allele frequency reported. The NGS assay employed was DNA-based and used a targeted panel covering 19 genes associated with lung cancer, including key oncogenic drivers such as EGFR, ALK, ROS1, and KRAS. Given brain metastases, we used entrectinib as a subsequent treatment at a dose of 600 mg per day (a potent oral multi-target TKI against ROS1, ALK, and NTRK-1, -2, and -3). According to the CT results on June 17th,2024, an efficacy assessment was performed and showed a partial response based on the Response Evaluation Criteria in Solid Tumors 1.1 criteria (**Figures 1G, H**). The brain metastases showed regression based on the June 17th, 2024 MRI (**Figures 2G, H**). Concurrently, notable reductions in the levels of tumor markers CEA and CA153 were observed during entrectinib therapy (**Figures 3A, B**). Presently, the patient remains stable and is willing to receive further treatment in our hospital (**Figure 4**).

## 3 Discussion

Herein, we reported a case of lung adenocarcinoma without driver alteration that developed resistance after multiple cycles of pembrolizumab treatment and newly emerged CD74-ROS1 fusion and achieved a partial response after entrectinib treatment. Of note, it is the first report of newly emerged ROS1 rearrangement after resistance to immune checkpoint inhibitors in clinical practice. Because of the rarity and thought-provoking nature of the case, we hope that the presentation of the case will offer insights to physicians and encourage the re-evaluation of driver genes in patients resistant to ICIs.

Currently, guidelines classify the first-line treatment of patients with advanced NSCLC into two primary approaches: targeted therapy and immunotherapy, determined by the presence of driver alterations. Pembrolizumab, an immune checkpoint inhibitor that targets the PD-1 (Programmed Death-1) receptor, is extensively utilized in the treatment of lung cancer, melanoma, and gastric cancer. For patients with adenocarcinoma exhibiting high PD-L1 expression (≥ 50%) and lacking driver gene mutations, pembrolizumab is recommended as a first-line monotherapy (10). Conversely, for those with low PD-L1 expression (< 50%), a combination of chemotherapy using carboplatin or cisplatin with pemetrexed alongside pembrolizumab is preferred (10). Unfortunately, nearly 70% of advanced NSCLC patients do not achieve lasting benefits from ICI-based therapies due to resistance to ICIs (11). This resistance arises from complex interactions among the host, tumor cells, and the immune microenvironment (12). Currently, the primary approach to addressing ICIs resistance is a combination therapy strategy, including combined with other types of immunotherapy drugs, chemotherapy, anti-angiogenic drugs, and radiotherapy. Among these, radiotherapy is considered a promising strategy for reversing resistance in NSCLC due to the “abscopal effect” (13, 14). For example, in the MDACC trial, stereotactic body radiation therapy (SBRT) was associated with increased treatment response rate and improved progression‐free survival (PFS) (15). Nevertheless, there remains a lack of consensus regarding optimal subsequent treatment and the best timing for patients who exhibit resistance to ICIs.

Currently, the majority of NSCLC patients with positive driver genes exhibit minimal responses to ICIs. Certain driver genes, such as EGFR mutations and ALK rearrangements, have emerged as significant exclusion criteria for the clinical application of ICIs. A global multicenter retrospective study, known as ImmunoTarget, explored the efficacy of ICIs in NSCLC patients harboring oncogenic driver genes (16). Among the cases studied, seven NSCLC patients harboring ROS1 rearrangement received treatment with ICIs monotherapy, resulting in an 83% rate of progressive disease. This finding suggests that pembrolizumab monotherapy is not effective in mitigating disease progression in NSCLC patients with ROS1 rearrangement, which is similar to our case. This conclusion not only provides partial insight into the patient’s resistance to pembrolizumab in the context of newly emerged ROS1 rearrangement but also supports that there was no initial missed diagnosis of ROS1, as NSCLC with ROS1 rearrangement shows a poor response to ICIs. In other words, the newly emerged ROS1 rearrangement in this case developed after multiple rounds of immunotherapy. Furthermore, it is important to consider why a new ROS1 variant emerged following pembrolizumab treatment, as acquired gene mutations frequently arise after targeted therapy rather than immunotherapy, such as the T790M mutation and MET amplification in the context of EGFR TKI resistance, as well as ROS1 point mutations associated with crizotinib resistance.

We proposed that tumor heterogeneity is the underlying reason for the secondary ROS1 rearrangement observed in patients. Following multiple rounds of treatment with pembrolizumab, tumor cells lacking ROS1 rearrangement may be effectively eliminated. Simultaneously, subpopulations of tumor cells with ROS1 rearrangement, which were not initially detected, may gradually expand and become the dominant clone within the tumor population due to their resistance to pembrolizumab, thus being discovered in subsequent testing. As noted above, there is currently no established treatment consensus for patients with lung adenocarcinoma who exhibit resistance to ICIs. Clinically, it is common practice not to retest driver genes in this patient cohort. Consequently, the critical takeaway from this case is the potential emergence of new therapeutic targets following long-term immunotherapy. We advocate for the retesting of driver genes in lung adenocarcinoma cases lacking driver alterations and exhibiting resistance to ICIs. Additionally, this finding may also hold significance for other histological subtypes of NSCLC.

There are some limitations in the present case. We hypothesize that the newly emerged ROS1 rearrangement occurs because the cell subset carrying ROS1 gradually becomes the predominant pathological type of adenocarcinoma following pembrolizumab treatment. However, there is currently a lack of basic research on this phenomenon, leaving its underlying mechanism unclear. Furthermore, the specific timing of when this cell subset becomes the primary component of the tumor remains unknown. Serial assessment of tumor mutational status may help address these open questions and guide subsequent treatment strategies. However, repeated invasive procedures like tissue biopsies can pose risks and discomfort to patients and increase healthcare costs. Additionally, obtaining a sufficient quantity of tissue for repeat genetic testing may prove challenging.

Briefly, we presented a case of a lung adenocarcinoma patient without driver alterations who developed resistance and a secondary ROS1 rearrangement following multiple rounds of pembrolizumab treatment. The patient subsequently achieved partial remission after receiving entrectinib. We propose that new therapeutic targets may emerge for this patient population following long-term immunotherapy, and we advocate for regular monitoring of tumor genetic status, which could yield unexpected benefits.

## Data Availability

The raw data supporting the conclusions of this article will be made available by the authors, without undue reservation.
